# The association between frailty risk and COVID-19-associated all-mortality in hospitalised older people: a national cohort study

**DOI:** 10.1007/s41999-022-00668-8

**Published:** 2022-06-24

**Authors:** Laia Maynou, Rhiannon Owen, Rob Konstant-Hambling, Towhid Imam, Suzanne Arkill, Deborah Bertfield, Andrew Street, Keith R. Abrams, Simon Conroy

**Affiliations:** 1 Department of Econometrics, Statistics and Applied Economics, Universitat de Barcelona, Avinguda Diagonal, Barcelona, Spain; 2 Department of Health Policy, London School of Economics and Political Science (LSE), Houghton St, London , WC2A 2AE UK; 3 Center for Research in Health and Economics, Universitat Pompeu Fabra, 23-25 Ramon Trias Fargas, Barcelona, 08005 Spain; 4 Swansea University, Swansea, UK; 5 NHS England, London, UK; 6 St Georges University Hospitals NHS Trust, London, UK; 7 University Hospitals of Leicester, Leicester, UK; 8 Royal Free London NHS Foundation Trust, London, UK; 9 London School of Economics, London, UK; 10 The University of Warwick, Coventry, UK; 11 University College London, London, UK

**Keywords:** COVID-19, Frailty, Acute hospital outcomes

## Abstract

**Introduction:**

Frailty has emerged as an important construct to support clinical decision-making during the COVID-19 pandemic. However, doubts remain related to methodological limitations of published studies.

**Methods:**

Retrospective cohort study of all people aged 75 + admitted to hospital in England between 1 March 2020 and 31 July 2021. COVID-19 and frailty risk were captured using International Classification of Disease-10 (ICD-10) diagnostic codes. We used the generalised gamma model to estimate accelerated failure time, reporting unadjusted and adjusted results.

**Results:**

The cohort comprised 103,561 individuals, mean age 84.1, around half female, 82% were White British with a median of two comorbidities. Frailty risk was distributed approximately 20% low risk and 40% each at intermediate or high risk. In the unadjusted survival plots, 28-day mortality was almost 50% for those with an ICD-10 code of U071 (COVID-19 virus identified), and 25–35% for those with U072 (COVID-19 virus not identified). In the adjusted analysis, the accelerated failure time estimates for those with intermediate and high frailty risk were 0.63 (95% CI 0.58–0.68) and 0.67 (95% CI 0.62–0.72) fewer days alive respectively compared to those with low frailty risk with an ICD-10 diagnosis of U072 (reference category).

**Conclusion:**

In older people with confirmed COVID-19, both intermediate and high frailty risk were associated with reduced survival compared to those with low frailty risk.

## Key summary points

**Aim:**

Frailty appears to be an important risk factor for COVID-19-related deaths, but studies to data have important limitations. The aim of this study was to describe the relationship between COVID-19, frailty risk and mortality in older people.

**Findings:**

This study examined outcomes for all older people hospitalised with COVID-19 between March 2020 and July 2021 and showed that mortality risk was increased with higher Hospital Frailty Risk Scores.

**Message:**

Any level of elevated frailty risk should be considered an important prognostic marker for older people hospitalised with COVID-19.

## Introduction

The COVID-19 pandemic has had a disproportionate impact upon older people, especially those with multiple comorbidities, such as chronic obstructive pulmonary disease, diabetes and dementia [1–3]. In addition to addressing comorbidities, an emerging feature of the clinical response has been to use the frailty construct to refine estimates of the risk of poor outcomes in the heterogeneous population of older people [4–6]. Frailty is a state of increased vulnerability to poor resolution of homoeostasis after a stressor event, which increases the risk of adverse outcomes in the acute care context, including delirium, disability and death [7–9].

Emerging evidence suggests frailty may be a predictor of poor outcomes in older people hospitalised with COVID-19 [10, 11]. However, the majority of studies that have examined the link between frailty and COVID-19 focussed upon selected samples (typically non-consecutive or convenience samples, or only those with frailty data available) of older people with COVID-19, rather than all older people admitted to hospital with suspected COVID-19 (the exception being Kundi et al. [12] from Turkey). This raises the possibility of selection bias. In addition, there were wide variations in the use of prognostically important covariates for risk adjustment: age and sex were used commonly, but comorbidities, ethnicity, socioeconomic status, previous hospitalisation, illness severity and time since the start of the pandemic (to reflect evolving treatments) were used in only a minority of reports [13]. This risks estimation bias. Moreover, many studies only captured in-hospital mortality, risking outcome bias. These limitations have important implications for clinicians using frailty as part of their ongoing COVID-19 risk assessment processes, as suggested by the National Institute of Health and Care Excellence [14].

Given these limitations with existing studies, and the potential impact upon clinical decision-making, we examined the relationship between frailty risk and 28-day mortality for all older people admitted to hospital in England and tested for COVID-19 between 2020 and 2021. To assess frailty risk, we employed the Hospital Frailty Risk Score (HFRS), which uses International Classification of Disease (ICD) codes generated following a hospital admission to estimate the risk of frailty and associated mortality. Although the HFRS is not a frailty index, it is moderately correlated with other frailty scales [15], includes ICD-10 codes that capture common frailty syndromes, such as falls, fractures, dementia and delirium, and has the advantage that it can be constructed for nearly everyone 75 years or older admitted to hospital.

The aim of this study was to examine the relationship between frailty risk and COVID-19-associated mortality using the Hospital Frailty Risk Score applied to the English population of older people admitted with COVID-19 between 2020 and 2021.

## Methods

### Design

This was a retrospective cohort study using the NHS England Secondary Uses Service (SUS) electronic database. A full description of the data linkage, security and processing is detailed in the study protocol [16].

The analysis used data relating to individuals’ first emergency presentation during which they received a COVID-19 test, between 1 March 2020 and 31 July 2021. Participants were included if they were 75 years or older and had an ICD-10 diagnostic code of U07.1 ‘COVID-19 virus identified’ or U07.2 ‘COVID-19 virus not identified’.

### Sample

The data represent all National Health Service (NHS) patients admitted to hospital with suspected COVID-19 in England. At the end of August 2021, there were 103,561 patients over 75 years of age who had been hospitalised and who had a COVID-19 test available for whom linkage was possible. Participants were only excluded if they did not have information essential to undertake linkage. Missing data were handled using the complete case analysis approach.

### Outcome variables

The primary outcome was all-cause mortality captured by the Office for National Statistics (ONS). We reported mortality at 28 days from the date of admission, consistent with NHS practice in England; individuals were censored if they were still alive at 28 days.

### Explanatory variables

COVID-19 status was defined by ICD-10 codes U07.1 ‘COVID-19, virus identified’ and U07.2 ‘U07.2 COVID-19, virus not identified’ recorded in the SUS record. U07.1 is used when COVID-19 has been confirmed by laboratory testing and U07.2 when a clinical diagnosis of COVID-19 was suspected, but where laboratory confirmation is inconclusive or not available. These groups were analysed separately in case there was some systematic difference in those with vs. without positive laboratory tests. For example, it is possible that people with delirium were not tested early on in the pandemic, as it took a whilst for delirium to be widely recognised as possible presenting feature of COVID-19 [17].

Frailty risk was measured using the HFRS, which uses ICD-10 codes to assign patients to three categories of low (< 5), intermediate (5–15), and high frailty risk (> 15) [15]. The HFRS uses ICD-10 codes pertaining elective or non-elective hospital admissions to generate a frailty risk score. The HFRS uses diagnostic information in an algorithm that identifies the risk of frailty and outcomes, such as death or unplanned hospital readmissions [15]. In the national validation cohort (*n* = 1,013,590), compared with the 42% patients with the lowest risk scores, the 20% patients with the highest HFRSs had increased odds of 30-day mortality (odds ratio 1.71; 95% CI 1.68–1.75), long hospital stay (6.03; 5.92–6.10), and 30-day readmission (1.48; 1.46–1.50). The HFRS offers an opportunity to assess frailty risk as a case-mix characteristic; its relative ease of application makes it an ideal tool for use in national datasets to provide a population perspective. We drew on diagnostic information from the patient’s current hospital admission and data from any previous emergency admissions that occurred within two years of the index admission to construct the HFRS [18]. We were principally interested in estimating the rate of death for those with and without confirmed COVID-19 and at different HFRS levels and thus we tested for an interaction between COVID-19 status and HFRS category.

The analyses controlled for differences in individual characteristics, using a broad set of risk adjusters including age, sex, ethnicity, deprivation (Index of Multiple Deprivation (IMD) quintile), Charlson Comorbidity Index [19], number of previous admissions, number of procedures, and Ambulatory Care Sensitive Conditions (ACSCs, defined as per the NHS Digital [20].

### Analyses

We used the generalised gamma model to estimate accelerated failure time as the proportional hazards assumption was violated. Estimated coefficients were reported as time ratios, illustrating an acceleration (coefficient > 1 indicating longer survival) or deceleration of survival time (coefficient < 1 indicating shorter survival). Model fit and comparison of accelerated failure time models was assessed using the Akaike Information Criterion (AIC) and Cox–Snell Residuals. Adjusted and unadjusted time ratios were used to compare the rate of death for those with and without confirmed COVID-19, at different levels of HFRS. We tested for an interaction between confirmed COVID-19 and HFRS status. All analyses were fitted in STATA version 16.

### Ethical and regulatory considerations

This study was conducted according to the current revision of the Declaration of Helsinki and ICH Guidelines for Good Clinical Practice. Fulham Research Ethics Committee (part of the UK Health Regulatory Authority) reviewed the application and issued a Favourable Opinion (reference 289267). The University of Leicester acted as study sponsor (reference 0804 COVID & Frailty).

## Results

Our sample comprised 103,561 observations with descriptive data summarised in Table 1.

**Table 1:** Baseline characteristics of all eligible participants at their index presentation, stratified by COVID-19 status

		U071 ‘COVID-19, virus identified’		U072 ‘COVID-19, virus not identified’	
Variable		(*n* = 93,325)		(*n* = 10,236)	
Age (mean, 95% CI)		84.1 (84.1–84.1)		84.1 (84.0–4.2)	
Female sex (*n*, proportion)		46,942 (50.3%)		4943 (48.3%)	
Hospital frailty risk score (*n*, proportion)	Low (< 5)	16,084	17.2%	1975	19.3%
	Intermediate (5–15)	34,556	37.0%	3867	37.8%
	High (> 15)	42,685	45.7%	4394	42.9%
Charlson comorbidity index (median, IQR)		2 (2)		2 (2)	
Ethnicity (*n*, proportion)	White	76,116	81.6%	8407	82.1%
	Asian	2372	2.5%	246	2.4%
	Afro-Caribbean	4639	5.0%	503	4.9%
	Other	10,198	10.9%	1080	10.6%
Index of multiple deprivation (IMD) score (mean, 95% CI)	First quintile	20,738	22.2%	2048	20.0%
	Second quintile	19,432	20.8%	2018	19.7%
	Third quintile	18,523	19.9%	2066	20.2%
	Fourth quintile	18,020	19.3%	2082	20.3%
	Fifth quintile	16,133	17.3%	1967	19.2%
Number of admissions in the previous 12 months (median, IQR)		1 (2)		1 (3)	
Number of procedures during the incident admission (median, IQR)		2 (4)		1 (4)	
Number presenting with an Ambulatory Care Sensitive Condition (n, proportion)		9200	9.9%	2145	21.0%

**Fig. 1:**
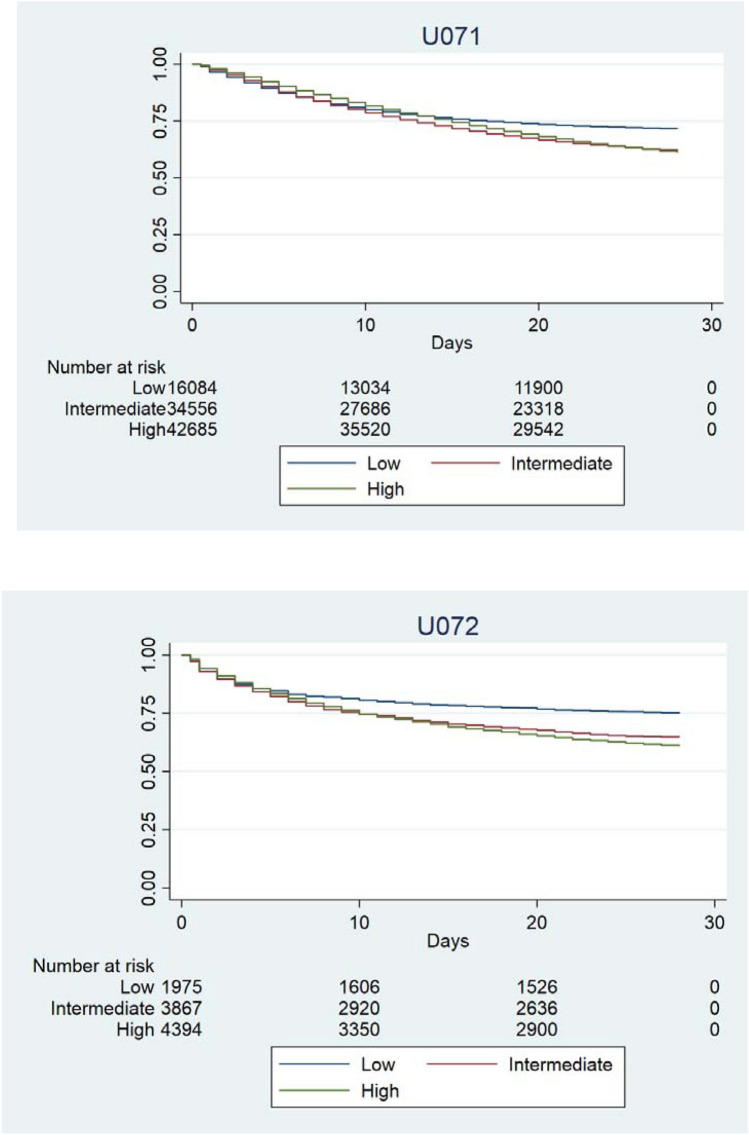
Kaplan–Meier plots showing time to all-cause deaths by HFRS category, stratified by ICD-10 code

Figure 1 shows the time to death by HFRS categories, stratified by COVID ICD-10 codes. In the unadjusted survival plots, 28-day mortality was almost 50% for those with an ICD-10 code of U071, and 25–35% for those with U072.

Table 2 shows the unadjusted and adjusted results, in which lower time ratios indicate a shorter survival time. An ICD-10 diagnosis code of U071 was associated with reduced survival time, estimated in the unadjusted analysis as 12.5% (time ratio (TR):0.88 (95% CI 0.78–0.98) fewer days alive, and 16% (TR: 0.84 (95% CI 0.76–0.93)) fewer days alive in the adjusted analysis.

**Table 2:** Unadjusted and Adjusted time ratios for all-cause mortality in hospitalised individuals with or without COVID-19

Main effects	Unadjusted *n* = 103,561			Adjusted *n* = 103,009		
	Time ratio	95% CI	*p*-value	Time ratio	95% CI	*p*-value
U072	Reference	Reference	Reference	Reference	Reference	Reference
U071	0.88	(0.78, 0.98)	< 0.001	0.84	(0.76, 0.93)	0.001
Low HFRS	Reference	Reference	Reference	Reference	Reference	Reference
Intermediate HFRS	0.82	(0.78, 0.85)	< 0.001	0.85	(0.81, 0.89)	< 0.001
High HFRS	0.90	(0.86, 0.94)	0.003	0.99	(0.95, 1.04)	0.704
Interaction effects						
Low HFRS* U071	Reference	Reference	Reference	Reference	Reference	Reference
Intermediate HFRS* U071	0.86	(0.76, 0.99)	0.029	0.88	(0.78, 1.0)	0.048
High HFRS* U071	0.79	(0.70, 0.90)	< 0.001	0.80	(0.71, 0.91)	< 0.001
Main effects for adjusted model						
Age (per year)				0.97	(0.97, 0.97)	< 0.001
Male				Reference	Reference	Reference
Female				0.69	(0.67, 0.71)	< 0.001
Charlson comorbidity Index = 0				Reference	Reference	Reference
Charlson comorbidity Index = 1				0.72	(0.69, 0.76)	< 0.001
Charlson comorbidity Index = 2				0.68	(0.65, 0.71)	< 0.001
Charlson comorbidity Index = 3 +				0.58	(0.56, 0.61)	< 0.001
Deprivation quintile 1				Reference	Reference	Reference
Deprivation quintile 2				1.06	(1.02, 1.10)	0.006
Deprivation quintile 3				1.04	(0.99, 1.08)	0.093
Deprivation quintile 4				1.07	(1.03, 1.12)	0.001
Deprivation quintile 5				1.15	(1.10, 1.20)	< 0.001
White British				Reference	Reference	Reference
Asian				0.81	(0.74, 0.88)	< 0.001
Afro-Caribbean				0.58	(0.55, 0.62)	< 0.001
Other				0.83	(0.80, 0.87)	< 0.001
Previous admission				1.0	(1.0, 1.0)	0.003
Number of procedure				1.10	(1.10, 1.10)	< 0.001
ACSC				1.18	(1.13, 1.23)	< 0.001

Reference categories, U072, Low HFRS, Low HFRS* U072, male, deprivation quintile 1, Charlson Comorbidity Index = 0, Previous admissions = 0 and White British

**Fig. 2:**
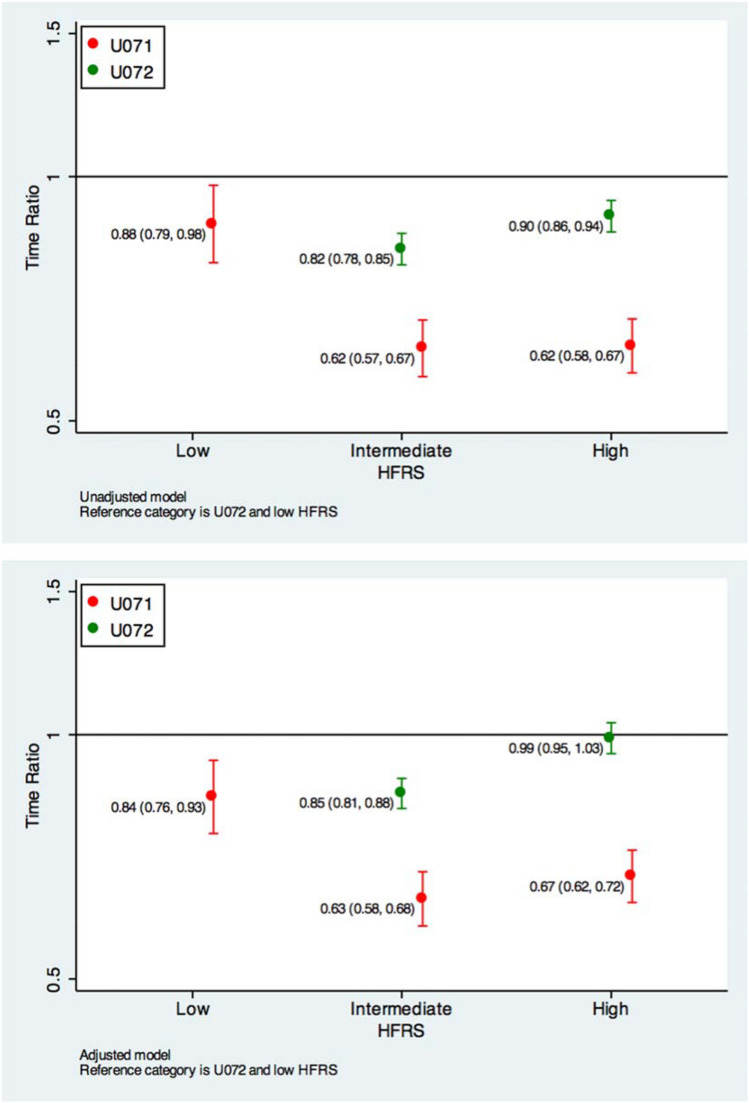
Time ratios for unadjusted and adjusted models

In the unadjusted analysis, those with an ICD-10 diagnosis code of U072 and intermediate frailty risk, had 18% (TR: 0.82 (95% CI 0.78–0.85)) fewer days alive, whilst those with high frailty risk had 11% (TR: 0.90 (95% CI 0.86–0.94)) fewer days alive compared to those with low frailty risk. In the adjusted analysis, the intermediate frailty risk appeared to have 15% (TR: 0.85 (95% CI 0.81–0.89)) fewer days alive compared to those with low frailty risk, whilst the estimated difference was not statistically significant for those with high frailty risk (TR: 0.99 (95% CI 0.95–1.03)) compared to those with low frailty risk (Fig. 2).

In the unadjusted analysis, those with either intermediate (TR: 0.62 (95% CI 0.57–0.67)) or high (TR: 0.62 (95% CI 0.58–0.67)) frailty risk with an ICD-10 diagnosis code of U071 had 38% fewer days alive, compared to those with low frailty risk with an ICD-10 diagnosis code of U072 (Fig. 2). In the adjusted analysis, the estimates for those with intermediate and high frailty risk were 37% (TR: 0.63 (95% CI 0.58–0.68)) and 33% (TR: 0.67 (95% CI 0.62–0.72)) fewer days alive compared to those with low frailty risk with an ICD-10 diagnosis of U072, respectively (Fig. 2).

As regards the influence of the adjustment factors, survival time was lower for older people, for women, for those from more deprived communities, and for people of non-White ethnicity. Survival time was higher for those who had more hospital procedures and if admitted with an Ambulatory Care Sensitive Condition (Table 2).

In view of the possible collinearities between the Charlson score and previous admissions with the HFRS, we undertook sensitivity analyses excluding these separately and in combination. The findings were robust to these sensitivity analyses; approximately 550 people had some missing IMD data. The distribution of the HFRS was approximately in keeping with previous reports [15, 21].

Figure 2 shows the time to death by HFRS categories, stratified by COVID ICD-10 codes. In the unadjusted survival plots, 28-day mortality was almost 50% for those with an ICD-10 code of U071, and 25–35% for those with U072.

Table 2 shows the unadjusted and adjusted results, in which lower time ratios indicate a shorter survival time. An ICD-10 diagnosis code of U071 was associated with reduced survival time, estimated in the unadjusted analysis as 12.5% (time ratio (TR):0.88 (95% CI 0.78–0.98) fewer days alive, and 16% (TR: 0.84 (95% CI 0.76–0.93)) fewer days alive in the adjusted analysis.

In the unadjusted analysis, those with an ICD-10 diagnosis code of U072 and intermediate frailty risk, had 18% (TR: 0.82 (95% CI 0.78–0.85)) fewer days alive, whilst those with high frailty risk had 11% (TR: 0.90 (95% CI 0.86–0.94)) fewer days alive compared to those with low frailty risk. In the adjusted analysis, the intermediate frailty risk appeared to have 15% (TR: 0.85 (95% CI 0.81–0.89)) fewer days alive compared to those with low frailty risk, whilst the estimated difference was not statistically significant for those with high frailty risk (TR: 0.99 (95% CI 0.95–1.03)) compared to those with low frailty risk (Fig. 2).

In the unadjusted analysis, those with either intermediate (TR: 0.62 (95% CI 0.57–0.67)) or high (TR: 0.62 (95% CI 0.58–0.67)) frailty risk with an ICD-10 diagnosis code of U071 had 38% fewer days alive, compared to those with low frailty risk with an ICD-10 diagnosis code of U072 (Fig. 2). In the adjusted analysis, the estimates for those with intermediate and high frailty risk were 37% (TR: 0.63 (95% CI 0.58–0.68)) and 33% (TR: 0.67 (95% CI 0.62–0.72)) fewer days alive compared to those with low frailty risk with an ICD-10 diagnosis of U072, respectively (Fig. 2).

As regards the influence of the adjustment factors, survival time was lower for older people, for women, for those from more deprived communities, and for people of non-White ethnicity. Survival time was higher for those who had more hospital procedures and if admitted with an Ambulatory Care Sensitive Condition (Table 2).

In view of the possible collinearities between the Charlson score and previous admissions with the HFRS, we undertook sensitivity analyses excluding these separately and in combination. The findings were robust to these sensitivity analyses.

## Discussion

After taking account of the interaction between HFRS and U071, intermediate and high frailty risk were associated with reduced survival compared to those with low frailty risk. However, there was minimal difference between intermediate and high-risk frailty in patients with a U071 diagnosis code. Other variables associated with reduced survival included an ICD-10 diagnosis code of U071, age, female sex, increasing comorbidities and non-White British ethnicity. Those undergoing procedures or presenting with ambulatory care sensitive conditions had increased survival compared to those without.

These findings support the previous studies of selected samples of older people with COVID-19 that show frailty is associated with reduced survival in people with COVID-19 [10, 11, 22]. However, an important finding from this analysis is that there appears to be relatively little separation in terms of survival when comparing those at intermediate and high risk of frailty, which supports some previous reports [23, 24] but not all [10, 22]. This has important implications for clinical practice, in which the presence of severe frailty (broadly HFRS scores > 15) is considered to be a marker of poor outcomes, and used to guide more supportive or palliative approaches, whereas those with moderate frailty (broadly HFRS scores between 5 and 15) would usually be considered for restorative care (should that be something that they value) [14]. Ambulatory Care Sensitive Conditions were associated with better survival, which has not previously been shown—these represent conditions that are considered preventable or could be treated in the community or an outpatient setting. These data could be used to identify cohorts suitable for home-based management of COVID, for example in the COVID virtual wards.

Important strengths of this study include the capture of all people aged 75 year or more admitted to all NHS hospitals with suspected COVID-19, using Routinely Collected data (RCD). RCD collected under real-world circumstances maximises representativeness of the study population and generalisability of findings, maximises resource efficiencies and allows the capture of information in large populations with continuously collected clinical events across long time periods [25]. We included a wide range of prognostically important covariates in the analysis to isolate the influence of frailty risk on mortality. We were able to report upon all-cause mortality both in-hospital and in the community, limiting the risk of outcome bias. It is possible that not all deaths reported here are directly related to COVID-19, particularly in the latter stages of the reporting period. However, in a range of sensitivity analyses looking at the impact of time from the start of the pandemic, we did not observe and significant changes in the rate of death nor the ability of the HFRS to predict deaths. We also included a wide range of covariates in our models that have not been widely reported in other studies yet are known to be important predictors of mortality: deprivation, previous admissions, and Ambulatory Care Sensitive Conditions. As with all studies that employ routine data, there is a possibility of coding error. However, especially given the heightened sensitivity about COVID-19 diagnoses and the medical examiner scrutiny that is part of the death certification process in England, this is unlikely to be a major threat to internal validity.

The HFRS is not a frailty score or scale, but a score to identify older people in whom frailty is likely to be prevalent. In the original validation study, the HFRS showed fair overlap with dichotomised Fried and Clinical Frailty Scale (CFS) (kappa scores 0.22, 95% CI 0.15–0.30 and 0.30, 0.22–0.38, respectively) and moderate agreement with the Frailty Index (Pearson's correlation coefficient 0.41, 95% CI 0.38–0.47). This is consistent with the performance of other frailty scores, with agreement between frailty ratings tending to be fair or moderate; kappa coefficients comparing Fried to Rockwood for example range from 0.3 to 0.5, depending on the measurement approach [26]. In the context of COVID-19, other studies have found a fair correlation between the CFS and the HFRS (Spearman *r* = 0.34) [27]. The benefits of using the HFRS is that it can be constructed for all those admitted to hospital using up-to-date diagnostic information from the current admission and previous admissions to generate frailty risk scores [18]. However, it is possible that in individuals with comorbidities for whom this was their first presentation to hospital, frailty risk might be underestimated. This might explain some of the overlap in survival between the intermediate and high frailty risk groups.

We demonstrated an interaction between frailty and an ICD-10 code of U071 that significantly increased the rate of mortality. However, there is little difference in the rate of mortality between high and intermediate HFRS. This may be related to changes in the nature of the population presenting as a consequence of the national lockdown [28], or the availability of testing, which changed over the course of the pandemic. Indeed, we observed both changing populations presenting to acute care over the course of the pandemic (in part related to the imposition and subsequent lifting of lockdowns) and changing outcomes (perhaps related to changing treatment effects, such as the introduction of Dexamethasone [29]). Other studies using population-level, representative datasets have confirmed the dynamic nature of the population presenting during the pandemic, and changes in treatments that have an important effect [21], which has not been addressed in the majority of studies reporting upon frailty and COVID-19 outcomes.

Intermediate or high frailty risk are similarly associated with reduced survival in older people with hospitalised with COVID-19; this extends the use of the frailty construct to alert clinicians to possible adverse outcomes. Frailty is likely to be a useful addition to risk prediction when used alongside other COVID-19 specific tools (e.g. 4C score [30]). Whichever risk scoring system is used, decisions about treatment should always be individualised, and incorporate shared decision-making involving patients and their advocates [14].
