# Correction: The association between frailty risk and COVID-19-associated all-mortality in hospitalised older people: a national cohort study

**DOI:** 10.1007/s41999-022-00684-8

**Published:** 2022-08-09

**Authors:** Laia Maynou, Rhiannon Owen, Rob Konstant-Hambling, Towhid Imam, Suzanne Arkill, Deborah Bertfield, Andrew Street, Keith R. Abrams, Simon Conroy

**Affiliations:** 1 Department of Econometrics, Statistics and Applied Economics, Universitat de Barcelona, Avinguda Diagonal, Barcelona, Spain; 2 Department of Health Policy, London School of Economics and Political Science (LSE), Houghton St, London , WC2A 2AE UK; 3 Center for Research in Health and Economics, Universitat Pompeu Fabra, 23-25 Ramon Trias Fargas, Barcelona, 08005 Spain; 4 Swansea University, Swansea, UK; 5 NHS England, London, UK; 6 St Georges University Hospitals NHS Trust, London, UK; 7 University Hospitals of Leicester, Leicester, UK; 8 Royal Free London NHS Foundation Trust, London, UK; 9 London School of Economics, London, UK; 10 The University of Warwick, Coventry, UK; 11 University College London, London, UK

## Correction: European Geriatric Medicine 10.1007/s41999-022-00668-8

The Funding information section was missing from this article and should have read ‘Laia Maynou is funded by the Spanish Ministry of Science, Innovation and Universities (PID2019-104319RB-I00)’.

The original article has been corrected.

